# Discrimination of normal and cancerous human skin tissues based on laser-induced spectral shift fluorescence microscopy

**DOI:** 10.1038/s41598-022-25055-y

**Published:** 2022-12-03

**Authors:** A. Niazi, P. Parvin, A. Jafargholi, M. A. Basam, Z. Khodabakhshi, A. Bavali, K. Kamyab Hesari, Z. Sohrabizadeh, T. Hassanzadeh, L. Shirafkan Dizaj, R. Amiri, O. Heidari, M. Aghaei, F. Atyabi, A. Ehtesham, A. Moafi

**Affiliations:** 1 Department of Physics and Energy Engineering, Amirkabir University of Technology, P.O. Box 15875-4413, Tehran, Iran; 2 Department of Electronic and Electrical Engineering, University College London (UCL), London, England, UK; 3 Faculty of Physics, Shahrood University of Technology, Shahrood, Iran; 4 Department of Dermatopathology, Razi Hospital, Tehran University of Medical Sciences, Tehran, Iran; 5 Department of Pathology, Razi Hospital, POX:1199663911, Tehran, Iran; 6 Department of Ocean Operations and Civil Engineering, Norwegian University of Science and Technology (NTNU), 6009 Ålesund, Norway; 7 Nanotechnology Research Center, Faculty of Pharmacy, Tehran University of Medical Sciences, Tehran, Iran; 8 Radiation Oncology Department, School of Medicine Washington University, St. Louis, USA

**Keywords:** Cancer, Optics and photonics, Physics

## Abstract

A homemade spectral shift fluorescence microscope (SSFM) is coupled with a spectrometer to record the spectral images of specimens based on the emission wavelength. Here a reliable diagnosis of neoplasia is achieved according to the spectral fluorescence properties of ex-vivo skin tissues after rhodamine6G (Rd6G) staining. It is shown that certain spectral shifts occur for nonmelanoma/melanoma lesions against normal/benign nevus, leading to spectral micrographs. In fact, there is a strong correlation between the emission wavelength and the sort of skin lesions, mainly due to the Rd6G interaction with the mitochondria of cancerous cells. The normal tissues generally enjoy a significant red shift regarding the laser line (37 nm). Conversely, plenty of fluorophores are conjugated to unhealthy cells giving rise to a relative blue shift i.e., typically SCC (6 nm), BCC (14 nm), and melanoma (19 nm) against healthy tissues. In other words, the redshift takes place with respect to the excitation wavelength i.e., melanoma (18 nm), BCC (23 nm), and SCC (31 nm) with respect to the laser line. Consequently, three data sets are available in the form of micrographs, addressing pixel-by-pixel signal intensity, emission wavelength, and fluorophore concentration of specimens for prompt diagnosis.

## Introduction

Carl Zeiss has realized the first fluorescence microscope^1^. Since then, various types have been developed to improve spatial resolution by upgrading the entire performance and significant modifications. Coherent light sources and digital processing techniques are also employed to obtain high-resolution images.

Today, in vivo fluorescence microscopy is a noninvasive technique with high spatio-temporal resolution. Confocal laser scanning microscopy (CLSM) has been developed as a potential diagnostic tool^2,3^, combining high-resolution optical imaging with depth selectivity. Recently, high-speed microscopy is used for the in-situ acquisition of histological images of living tissues, and real-time high-resolution imaging is introduced for in-vivo skin cancer diagnosis^4–11^. The vital feature is capturing well-focused images from different sample depths based on optical sectioning^12–14^. The inherent diffraction limit of the image drastically restricts the spatial resolution up to $\sim$ 10 μm^15–17^, not enough to reveal the cell structures in detail. An interferometric technique called structured illumination microscopy (SIM) has been introduced to improve lateral resolution^18–23^. The photoactivated localization microscopy (PALM)^24^, stochastic optical reconstruction microscopy (STORM)^25^, and fluorescence photoactivation localization microscopy (FPALM) have been developed to achieve the highest ever spatial resolutions^26^.

Polarimetry discriminates benign tissues from cancerous skin lesions based on Mueller matrix elements, including diattenuation, depolarization, and retardance parameters. For instance, regarding the nucleus to cytoplasm ratio (N/C), the malignant lesions benefit from a smaller depolarization against the benign nevus^27,28^.

The identification of melanoma has been recently reported using laser-induced breakdown spectroscopy (LIBS)^29–31^. The line intensities of several biomarkers in the spectra exhibit a significant difference between melanoma and normal tissues. The elevated Ca, Mg, and Na trace elements exist in melanoma lesions, emphasizing the Mg regulating effect on cell division. However, LIBS demonstrates a poor ability to diagnose various malignant tissue types in detail^32–37^.

On the other hand, the spectral shift assessment of the fluorescence photons is crucial to diagnose neoplasia which mainly arises from different pathways of Rd6G interactions with malignant/healthy cells^38–43^. The fluorescence lifetime spectroscopy also demonstrates the competence for in-vivo cancer diagnosis^39^. Some inherent fluorescence properties of chemo drugs have been examined^44–47^ to distinguish normal/cancerous tissues^48–50^. Moreover, the multispectral fluorescence imaging system (MFIS) improves the resolution of micrographs^51^.

The American Academy of Dermatology Association (AAD) reports that ~ 206,000 new cases of melanoma, i.e., ~ 100,000 noninvasive and ~ 106,000 invasives, were diagnosed in the US in 2021. Invasive melanoma is the fifth most commonly diagnosed cancer for men and women. Here a homemade SSFM is fabricated to identify various malignant skin lesions. Despite the fluorescence microscope relying on the signal intensity, SSFM carefully deals with the spectral response of the stained tissues. According to the spectral shift measurements, the instrument’s performance is examined in favor of various normal/cancerous specimens^52^. The apparatus efficiently discriminates the malignant cases with a high confidence level. The spectral discrepancy arises from the abundance of Rd6G bondings to the cell mitochondria. Three fluorescence images are taken into account to map the specimen’s signal intensity, fluorescence emission wavelength, and the corresponding Rd6G concentration. The conjugates cause to obliterate the initial fluorophore population leading to distinct spectral shifts. The latter represents a unique property for tissue identification. Furthermore, RGB color codes address these spectra in sequential order to discern melanoma, basal cell carcinoma (BCC), and squamous cell carcinoma (SCC) in healthy or nevus specimens. In comparison, the intensity color codes undergo intense emission in healthy and relatively faint signals in nonmelanoma lesions. The Rd6G chemical affinity to cell conjugation activates different pathways favoring various lesions. The peak emission wavelengths demonstrate certain red/blue shifts to discriminate the lesions from normal tissues by vitrue of SSFM whose potential effect on early diagnosis of malignant cells is deeply investigated here. We have shown that SSFM is a promising tool for future in-vivo examination using biocompatible flourophores without need of biopsy.

## Results

### Bare fluorophores

The spectral shift addresses the change in emission wavelengths at the fluorescence signal’s peak. Usually, there is a redshift to longer wavelengths in terms of Rd6G concentration due to reabsorption events, whereas a blue shift takes place to shorter wavelengths when concentration reduces mainly due to the conjugation, complex formation and etc., leading to the obliteration of fluorophores. This means the changes in the wavelengths of the maximum fluorescence intensity of the total registered spectrum, which reflects both the fluorescent, absorbing and scattering properties of the components of the sample.

**Figure 1:**
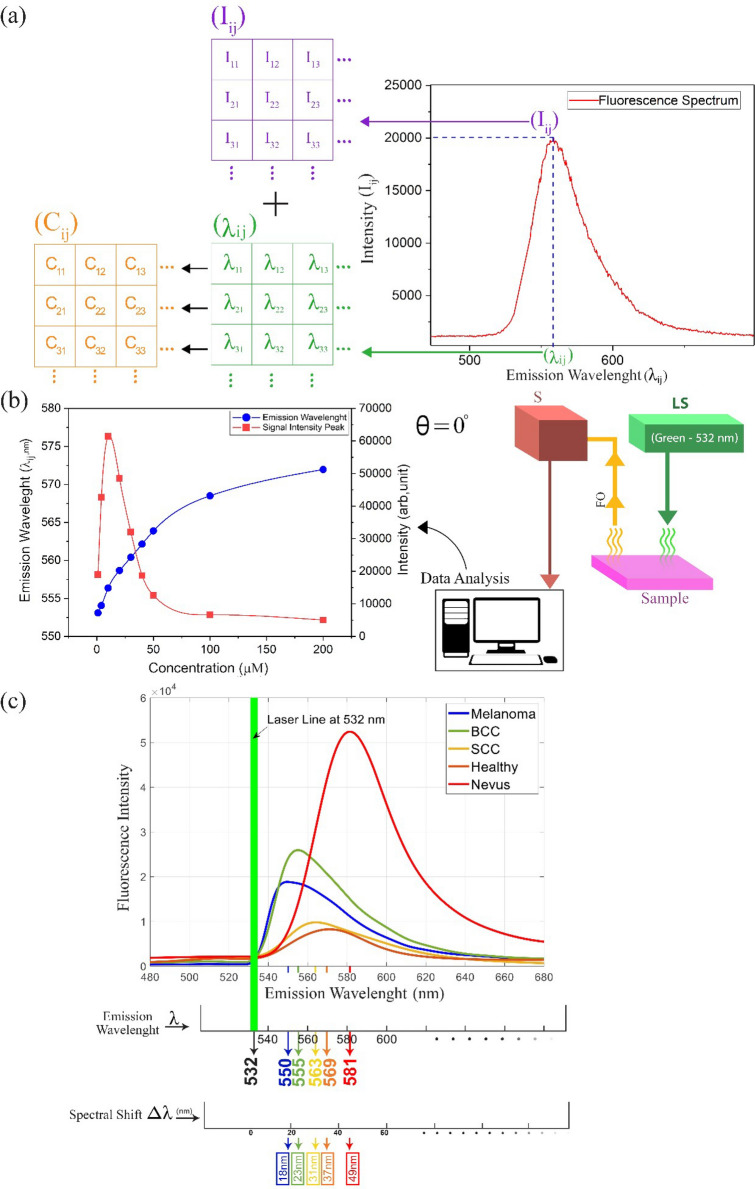
(**a**) Schematics of fluorescence emission micrographs based on m × n pixels. Each pixel owns its fluorescence spectrum, including max signal magnitude at a specific emission wavelength peak. After noise mitigation and image processing, the intensity and emission wavelength matrices lead to the tissue mapping. (**b**) Left: Emission wavelength and signal intensity versus Rd6G concentration ($\mathrm{\mu M})$ at the parallel incident. Right: laser-induced fluorescence (LIF) spectrometry at the parallel incident arrangement. Note that the spectra taken from the parallel arrangement are slightly different from the right-angle array. (**c**) Typical spectra and the corresponding spectral shift of nonmelanoma/melanoma lesions and normal/nevus tissues. The melanoma covers 550 $\pm$ 2 nm spectral shift (with respect to laser line) typically denotes 18 nm, nonmelanoma ranges 553–565 nm where BCC takes 555 $\pm$ 3 nm (23 nm), SCC corresponds to 563 $\pm$ 3 nm (31 nm). Similarly, normal tissues include an emission wavelength $\sim$ 570 $\pm$ 3 nm (37 nm), and nevus involves a wide range of emission wavelengths longer than 575 nm having 10 nm width (49 nm). Rd6G, Chemical formula, C_28_H_31_N_2_O_3_Cl, Molar mass,479.02 g/mol, Density, 1.26 g/cm^3^, Solubility in water, 20 g/l (25 °C), Absorption peak 530 nm, Emission spectra 540–560 nm. Here, the parallel arrangement is used in SSFM such that *S* spectrometer, *LS* laser source, *OF* optical fiber.

At first, the bare Rd6G properties are investigated where no tissue is stained. Figure 1a shows a typical fluorescence spectrum characterized by ${I}_{ij}$ and ${\lambda }_{ij}$ at pixel location (i,j), which ascertain the peak of the fluorescence signal and the corresponding emission wavelength at max intensity, respectively. Similarly, there is ${c}_{ij}$ distribution addressing the corresponding fluorophore concentration at the position (i,j). A strong correlation appears between ${c}_{ij}$ and ${\lambda }_{ij}$, more pronounced than the signal intensity by itself. Subsequently, the experimental measurements of the bare fluorophores are carried out in various concentrations. Figure 1b illustrates ${\lambda }_{ij}$ and ${I}_{ij}$ versus Rd6G concentration at the parallel incident arrangement ($\uptheta =0)$. Rd6G exhibits a fluorescence emission ranging from 540 to 600 nm, where the emission peak is redshifted at dense concentrations. Note that the parallel configuration is used as the optical setup of the proposed SSFM. In comparison, the right angle arrangement has been previously carried out whose spectra demonstrate a minor discrepancy against parallel configuration^53–55^.

### Fluorophore with tissue staining

The experiments are carried out after staining the tissues of interest with the Rd6G solution. The imaging technique is based on the target scanning indicating the pixel by pixel laser illumination and subsequent spectral detection. Figure 1c depicts various typical fluorescence spectra of the stained tissues. This delineates the key spectral shifts characterizing the distinct skin neoplasia. The signal intensity and the corresponding emission wavelength vary according to the tissue’s pathological/morphological properties. This causes distinct spectral red/blue shifts in conjunction with the various cancerous lesions. These are measured for melanoma (~ 18 nm), BCC (~ 23 nm), SCC (~ 31 nm) lesions, as well as typical normal (~ 37 nm) and nevus (~ 49 nm) skin tissues.

Note that the original spectral emission lies over 548–575 nm indicating optically green-yellow emissions. However, the algorithm implemented here extends this spectral range as blue-red color codes (Supplementary Table S1) to demonstrate the highest resolution favoring various malignant lesions alongside better visual identification. In fact, these spectral data are analyzed through software and translated into corresponding micrographs to facilitate the diagnosis visually.

The spectral shift addresses the change in emission wavelength at the fluorescence signal’s peak. Usually, there is a redshift in terms of Rd6G concentration due to reabsorption events, whereas a blue shift in the shorter wavelength takes place when concentration reduces mainly due to the conjugation, complex formation, or the interaction with cells leading to the obliteration of fluorophores. This means the changes in the wavelength of the maximum fluorescence intensity of the total registered spectrum reflect both the fluorescent, absorbing, and scattering properties of components of the sample.

**Figure 2:**
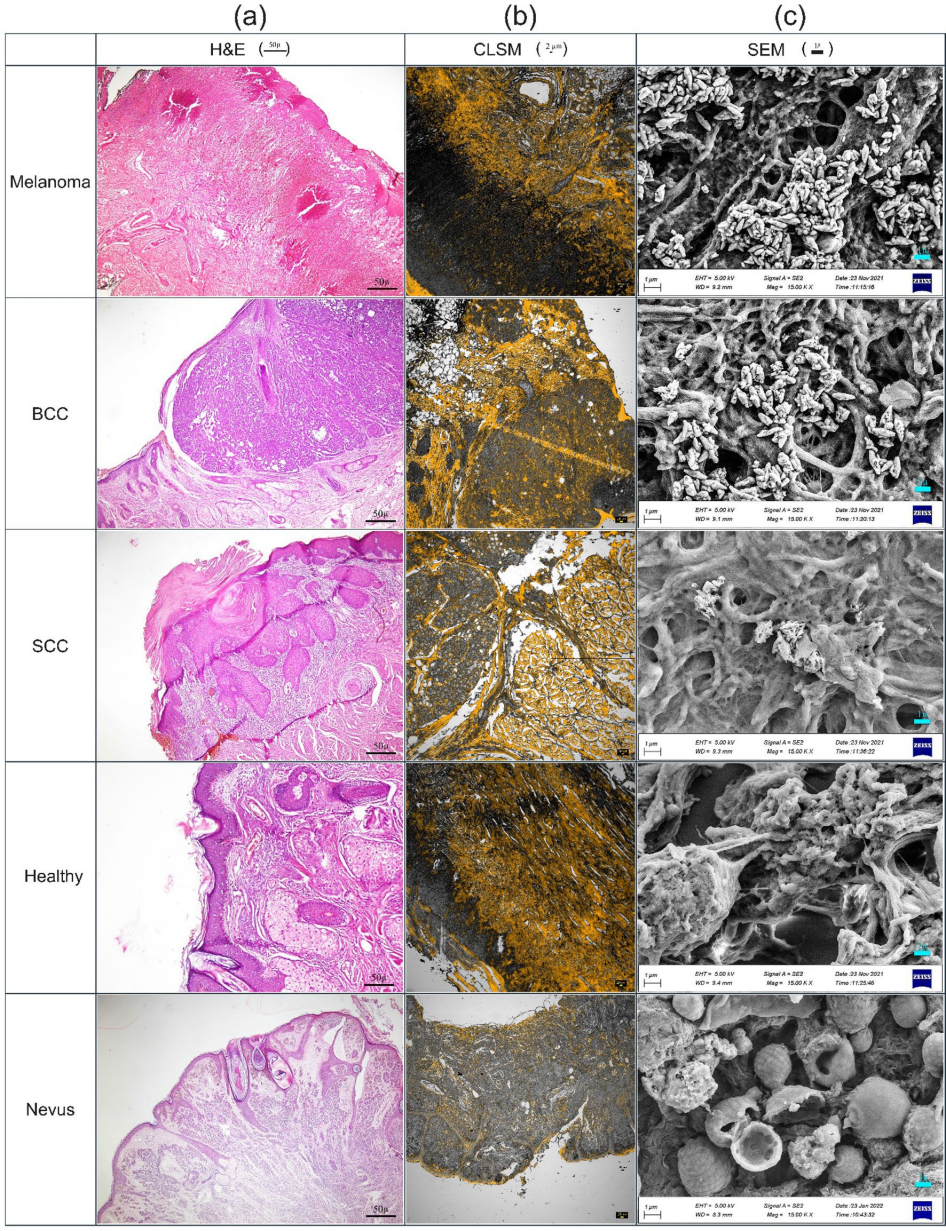

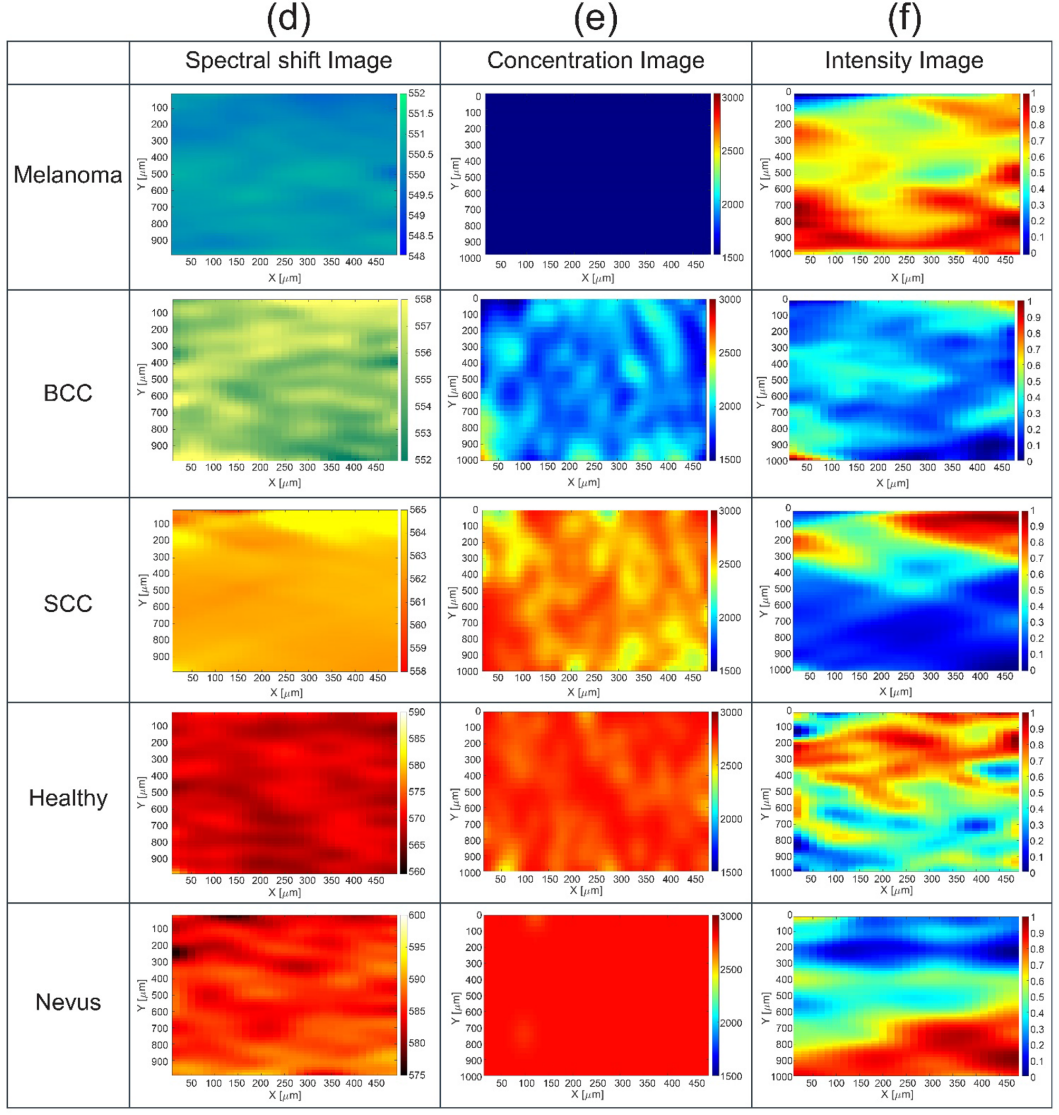
(**a**) Typical hematoxylin–eosin optical micrographs of melanoma, BCC, SCC, benign nevus, and normal skin tissues. (**b**) Corresponding CLSM indicates high-resolution images, including fluorophore distribution in yellow color. (**c**) Corresponding FE-SEM micrographs highlighting the local Rd6G conjugations. Typically SSFM micrographs are illustrated as (**d**) spectral shift, (**e**) Rd6G concentration distribution, and (**f**) signal intensity, respectively. Note that spectral shift images take dominant color codes of blue, green/yellow, and yellow/orange having Melanoma, BCC, and SCC, respectively. The normal tissues take vivid red, and nevi are characterized by dark red.

Figure 2a illustrates the H&E optical micrographs (scale 100 μm) of various neoplasic/normal tissues. The cytoplasm C looks pink due to its basicity, whereas nucleus N takes bluish regarding its acidity. Melanoma specimens benefit from a relatively lower N/C ratio, leading to mild pink. Furthermore, these cancerous cells form noncohesive nests, and pagetoid spreads without cell maturity in the dermis against nonmelanoma skin cancers. As the tumor progresses, the nest shows mitotic activity. On the other hand, BCC demonstrates a relatively higher N/C ratio^28^, giving a deep blue appearance. The basaloid nest shows a preferable palisade alongside the apparent clefts. Moreover, SCC appears pinkish due to its relatively lower N/C ratio, whose nuclei usually show more pleomorphism and atypia than BCC. Another characteristic feature is the intercellular bridges and the generation of keratin within the reddish dyskeratotic cells or in keratin pearl. On the other hand, normal basal and squamous cells are highlighted in healthy tissues’ epidermis. In benign melanocytic nevi, the cells become slender and spindled with less melanin pigment as they migrate from the upper to deep dermis.

Figure 2b shows the corresponding CLSM images (scale 2 μm), indicating the fluorophore distribution amid the cell structures. CLSM reveals no mitotic figure in basaloid or squamoid nests. According to the pathological criteria, the micrographs do not help diagnose tumors in oncology studies. CLSM takes high-resolution images alongside SSFM spectral micrographs. Figure 2c demonstrates neoplasia’s corresponding field effect-scanning electron microscope (FE-SEM) (scale 1 μm) versus normal tissues.

Subsequently, Fig. 2d–f are taken in virtue of SSFM to illustrate micrographs of the spectral shift, fluorophore concentration, and intensity of fluorescence emission over various cancerous lesions against healthy/nevi specimens, respectively. The color codes introduced in Supplementary Table S1 (view the supplementary information, extended data section) facilitate the physician’s diagnostic decision. Figure 2d illustrates a melanoma spectral shift micrograph with dominant blue color, BCC approaches green/yellow, and SCC typically looks yellow/orange. Similarly, the normal tissues also take abundant reddish, and nevus resembles dark red. The invasive melanoma contains a heavy cellular population accompanying ample fluorophore bondings to the mitochondria, where blue color code appears in spectral images equivalent to $\sim$ 18 nm shift ($\sim$ 550 nm) with respect to the laser line at 532 nm. The notable blue shift appears due to the formation of conjugation and bondings in cancerous tissues. The quenching of excessive active fluorophores agrees with the (Rd6G + malignant cell) phenomena. The emission wavelength mapping on behalf of BCC lesions exhibits a significant green/yellow color code, emphasizing a spectral shift of 23 nm shift against the laser line. The BCC emphasizes a 5 nm shift to a longer wavelength (555 nm) with respect to melanoma micrographs. SCC undergoes more populated active fluorophores against BCC micrographs whose emission wavelength attest to be 563 nm, equivalent to 31 nm redshift against laser line, emphasizing a certain redshift of 8 nm in the spectral micrographs regarding BCC.

Normal tissues undergo a vivid red, where a slight number of fluorophore conjugations are congested around the area of the cell regarding the low affinity of Rd6G chemical interaction. In addition, benign nevi benefit no obliteration of active fluorophores leading to the longest emission wavelength as dark red. Furthermore, cancerous tumors deform and terminate the collagen fibers^28^, so the cancerous lesions suffer collagen destruction creating active sites for Rd6G attachments regarding normal tissues. As a result, a practical way is proposed to identify the degree of nonmelanoma neoplasia. The abundance of fluorophore bondings notably varies in the cancerous structures leading to a drastic blue shift for the lethal malignant tissues against the healthy ones. The better performance of spectral shift assessment is realized using SSFM mainly due to negligible parasitic effect and inherent denoising properties.

Due to concentration assessments, RGB is set to assign a range of 0.1–200 μM, such that R attributes dense and B belongs to dilute fluorophore populations. Figure 2e depicts the intensity images representing signal intensity mapping of the specimens. It displays a diversity of RGB distribution mainly due to the nonlinear function of emission wavelength with concentration, as shown in Fig. 1b. At the same time, the larger concentrations may lead to intense signal R or faint emission B related to the different quenching pathways. However, signal intensity generally attests to more intense emission of benign and normal tissues than nonmelanoma fluorescence emissions. The intensity micrographs in normal tissues include a variety of RGB distributions because of intense/faint emissions. The molecular conjugates obliterate the active fluorophores, leading to faint signal emissions and a larger blue shift against normal tissues.

Figure 2f illustrates the micrographs highlighting Rd6G concentrations over the tissue of interest based on the color codes, which copy the spectral features of micrographs according to Fig. 2d. As a result, Melanoma, BCC, SCC, and nevi/healthy tissues take dark B, mild B, yellow/orange, orange-reddish, and dominant red orderly with corresponding concentrations of 2, 8, 10, 12, and 16 μM, respectively. Despite the dense active fluorophore content in normal tissues, melanoma lesions take a relatively dilute concentration alongside highly conjugate formation.

## Discussion

Skin is the largest organ of the body, having many functions, including protective, sensory, thermoregulatory, metabolic, and sexual signaling. Histologically, it is composed of epidermis and dermis and overlies hypodermis. The epidermis consists of stratified squamous epithelium composed mainly of keratinocytes, melanocytes, langerhans, and merkel cells. The dermis is a layer of connective tissue that consists of the papillary and the reticular dermis. It also contains hair follicles, sweat glands, and different sensory receptors. Melanoma and nonmelanoma are known as the most frequent malignant tumors among whites.

BCC is the most common malignancy, found predominantly on the sun-exposed skin of the elderly. Microscopically, it includes islands and nests of basaloid cells. Peripheral palisading of tumor cells and clefting at the tumor-stroma interface are noted where the stroma may be myoxid. Sometimes calcification and amyloid deposition is observed in BCC. Various histological subtypes have also been defined, including nodular, micronodular, superficial multifocal, adenoid, infiltrating, sclerosing, keratotic, infundibulocystic, metatypical, basosquamous, and fibroepitheliomatous.

On the other hand, SCC is the second most common skin malignancy. It mainly occurs in sun-exposed areas of skin among older people. Histopathological examination reveals nests of squamous cells in the dermis connected with the epidermis. The tumor cells have abundant eosinophilic cytoplasm and larger vesicular nuclei. Sometimes keratinization and keratin pearl formation is noted in the center of the nests. The tumors can be divided into well, moderately, and poorly differentiated groups.

In comparison, malignant melanoma accounts for the majority of mortality due to skin cancer. The most common histologic features include superficial spreading melanoma, lentigo malignant, acral lentiginous, and nodular melanoma. Generally, some microscopic findings in melanoma include ill-defined borders, the pagetoid spread of melanocytes, epidermal consumption or ulceration, and architectural and cytological atypia in the junctional nests. The dermal component may also be noted, whether involving the papillary dermis or extending beyond it.

Photo-micrographs are usually taken using a fluorescence microscope, and CLSM images are also employed to diagnose cancerous cells^26^. Dermatologists almost encounter suspicious cancerous tissues with subtle pathological discrepancies. The diagnosis of tissue may be virtually challenging. Hence, the proposed SSFM can be coupled with a CLSM as a valuable accessory unit to improve the prompt diagnosis of lesions.

The fluorophore bindings to mitochondria occur at the attendance of Rd6G molecules around the cell, thus reducing the entire activated fluorophore population inhibiting mitochondrial metabolic activity. Subsequently, the respiratory chain functioning becomes blocked, and the cell would suffocate and eventually be destroyed. Rd6G-induced active mitochondria would selectively kill malignant but spare normal cells. Unlike normal, the malignant ones demonstrate notable reduced mitochondrial numbers alongside aberrant metabolism. In other words, melanoma undergoes rapid cell growth while the fluorophores are attached to ample mitochondria, damaging the entire cells. Note that the malignant cells are selectively demolished at low Rd6G doses, whereas the healthy ones may notably survive^56^.

**Figure 3:**
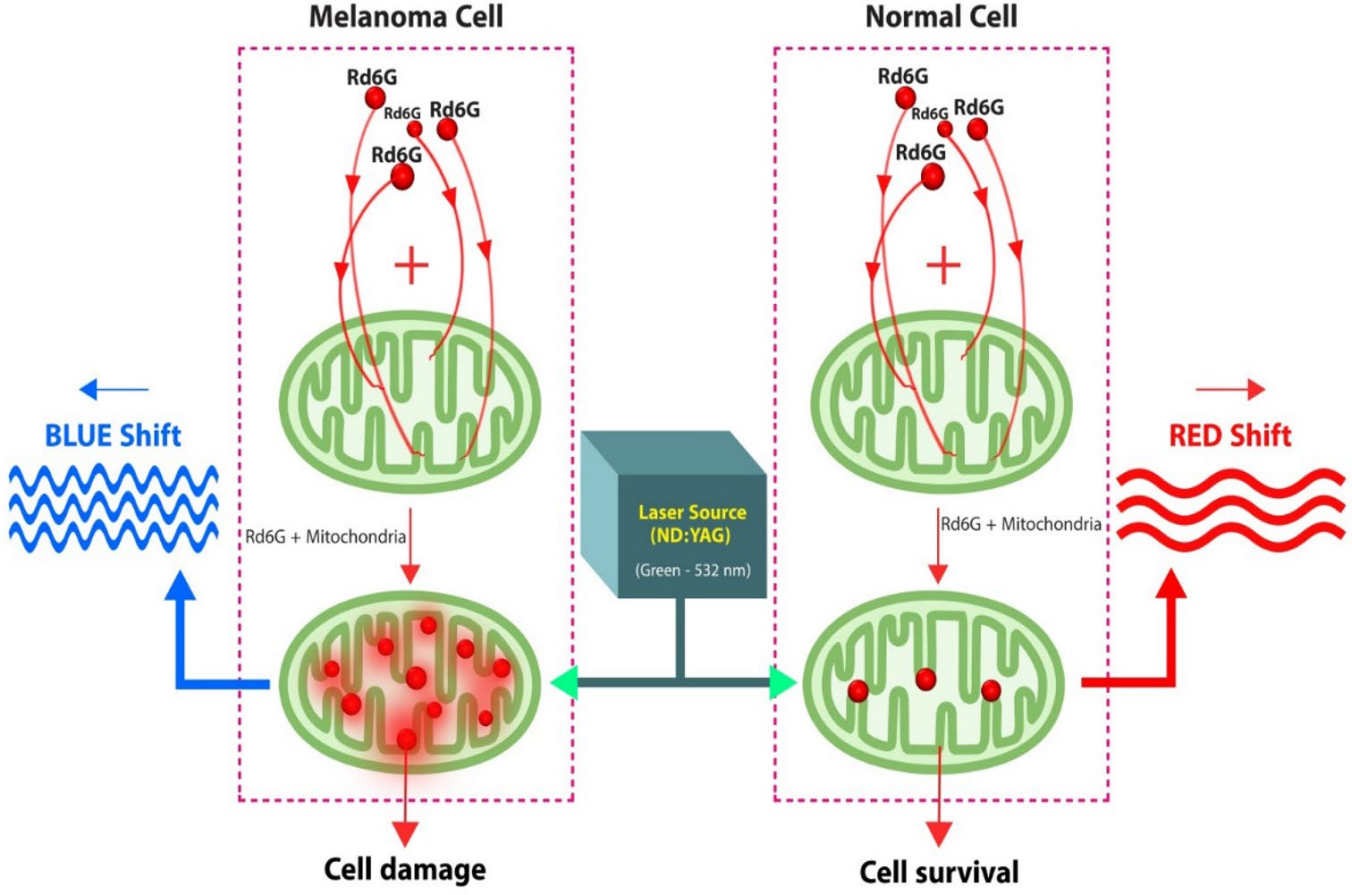
A conceptual model based on the graphical representation of (Rd6G + mitochondria) in melanoma and normal cells. After laser excitation, the stained melanoma undergoes a lucid blue shift, while the fluorescence emission of normal cells reveals redshift events. This key optical discrepancy is used to discriminate different lesions. Furthermore, unhealthy cells are most likely demolished after Rd6G exposure to mitochondria based on their ample conjugation and bindings, whereas normal cells survive due to low chemical affinity. This conceptual model is verified by virtue of statistical processing given in Supplementary (“Statistical analysis” section).

The healthy mitochondria also derive most adenosine 5ʹ-triphosphate (ATP) by metabolizing the consumed glucose to carbon dioxide and water, but the malignancy directly gives out lactic acid affecting the interaction with the fluorophores. Figure 3 displays a conceptual model based on the graphical representation of Rd6G interaction with normal/cancerous skin tissues according to the schematic function of Rd6G + mitochondria. Rd6G conjugation in melanoma tissues is much more pronounced, giving rise to cell damage alongside a relatively blue-shifted fluorescence emission. Conversely, the rare attachments with healthy cells lead to their survival with a characteristic redshift. Similarly, the nonmelanoma lesions are located in the middle because a relatively smaller number of bindings are involved^40–43^.

Stokes shift is the spectral separation between the excitation and emission, taken into account as one of the essential properties of the fluorophores. This addresses a narrow spectral spacing for most antitumoral fluorophores to change the emission wavelength maxima against the concentration. In general, the spectral shift arises from the propagation of photons in the material due to several events such as self-absorption, reabsorption, as well as fluorophore aggregation/bondings to the cells and the surrounding molecules. These events may vary the wavelength peak, a valuable property used to form the spectral fluorescence images. The spectral shift due to the fluorophore population is considered a diagnostic measure in highly scattering media (human tissues). It is shown that the signal intensity measurements do not provide adequate information regarding the tissue structure. It sometimes may scale up noise, leading to undesired false signals or low signal–noise ratio as dilute/dense fluorophore contents may give out similar emission signals.

Here, a method of optical imaging based on the spectral shift assessment is implemented^52^. The method includes generating a sample by mixing an object with a fluorophore, stimulating the sample by emitting a laser beam, extracting a plurality of fluorescence spectra from various fluorescence emissions emitted from the sample pixel by pixel, detecting the fluorescence peaks and the corresponding wavelengths of the spectra, extracting the fluorophore concentrations from the database, and eventually generating a concentration image based on emission wavelength image. The concentration and emission wavelength images address the specified pixels.

Various malignant human lesions are examined against the normal tissues. At first, the specimens are stained in Rd6G solution at a certain concentration. Then pixel by pixel laser-induced fluorescence (LIF) spectra is collected by scanning the area of interest. The normal stained tissues undergo a lucid redshift (relative to the laser line at 532 nm) versus Rd6G concentration, whereas the stained nonmelanoma demonstrates a relatively blue shift against healthy ones indicating a small redshift with respect to the laser line regarding the type and degree of neoplasia. It is worth noting that the dermatologist may make a hard decision due to the significant commonalities and subtle dissimilarities in the nonmelanoma cases, so SSFM may act as a valuable but rather inexpensive instrument to carefully differentiate suspicious skin lesions to resolve the correct decision. Furthermore, the color code determines the fluorophore distribution over a certain spectral emission of $\sim$ 25 nm, segmenting each type by certain spectral width. The color code distribution of a typical normal micrographs looks reddish. This notably shifts from red to yellow/green and green/blue, favoring SCC and BCC specimens discriminating in several distinct widths of 5 nm, respectively. Regarding the melanoma lesions, the color code resembles blue in comparison. Simultaneously, ${I}_{ij}$ magnitude fluctuates in conjunction with the red/blue shifts. Each pixel contains an emission peak having a corresponding signal amplitude, altering from one pixel to another. In other words, Rd6G molecular bindings with mitochondria of cancerous lesions are notably high to form plenty of conjugates leading to faint signals. In contrast, normal tissue features dense active fluorophores and intense emissions. Similarly, a couple of typical pixels with diluted and dense Rd6G concentrations may exhibit similar signal intensity. Normal tissues may show strong signals because of ample active fluorophores; however, faint signals arise from the quenching effects at dense concentrations. The normal tissue enjoys a partitioning effect to prevent the aggregation or resonance energy transfer (RET) effect^49^. Melanoma experiences more binding, giving rise to less active fluorophores content, indicating faint (blue) signals accordingly. Thus, strong signals most likely appear in median concentration. In addition, SSFM acquires the fluorescence data, including signal intensity I_ij_ and the emission wavelength $\lambda$_ij_, to reconstruct $\mathrm{C}$_ij_ spatial micrographs.

**Figure 4:**
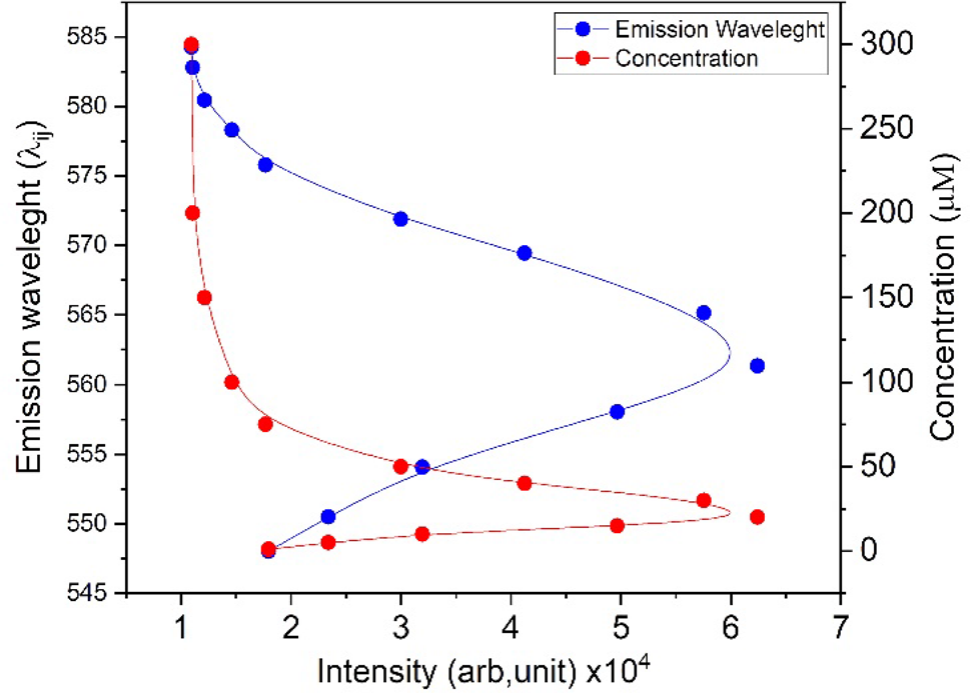
Emission wavelength and Rd6G concentration in terms of fluorescence signal intensity.

Figure 4 depicts Rd6G emission wavelength (548–575 nm) and the corresponding concentration (0–200 μM) in terms of signal intensity. This notably attests to a couple of values of emission wavelengths for a single signal intensity, hence unable to determine the true concentration generally. Thus, the signal intensity cannot map the accurate concentration while the emission wavelength does. For instance, intense signals may correspond to 10 or 40 μM. Therefore, intensity mapping may be necessary but not sufficient to determine the local Rd6G distribution differentiating dilute/dense concentrations in practice. We conclude that the redshift takes place because of a dense population. The emission shifts to a larger wavelength, according to Fig. 1b (main text), in the case of normal tissues. On the contrary, for malignant cells, Rd6G fluorophore interacts with cells (most likely mitochondria) to enhance the formation rate of conjugation, and a reduction of fluorophore concentration takes place, which gives rise to a lucid blue shift (emission shift to the shorter wavelength). We have shown that the rate of blue shift is much larger for melanoma tissues than for BCC and SCC specimens. Note that according to Fig. 1b, the emission wavelength versus concentration is nonlinear, and for this reason, there is a slight difference between the emission wavelength and concentration images. The various Rd6G concentrations (bare without tissues) were prepared initially, and the fluorescence signals (and emission wavelength at the peak of fluorescence signal) were recorded using the SSFM as shown in Figs. 1b and 4. The calibration curve—Fig. 4—lucidly correlates fluorescent signal/emission wavelength to the concentration. According to the main concept of this manuscript, the spectral shift is a more reliable method to determine the concentration than the fluorescence signal. Note that the relationship between [c] and [λ] is not linear.

**Figure 5:**
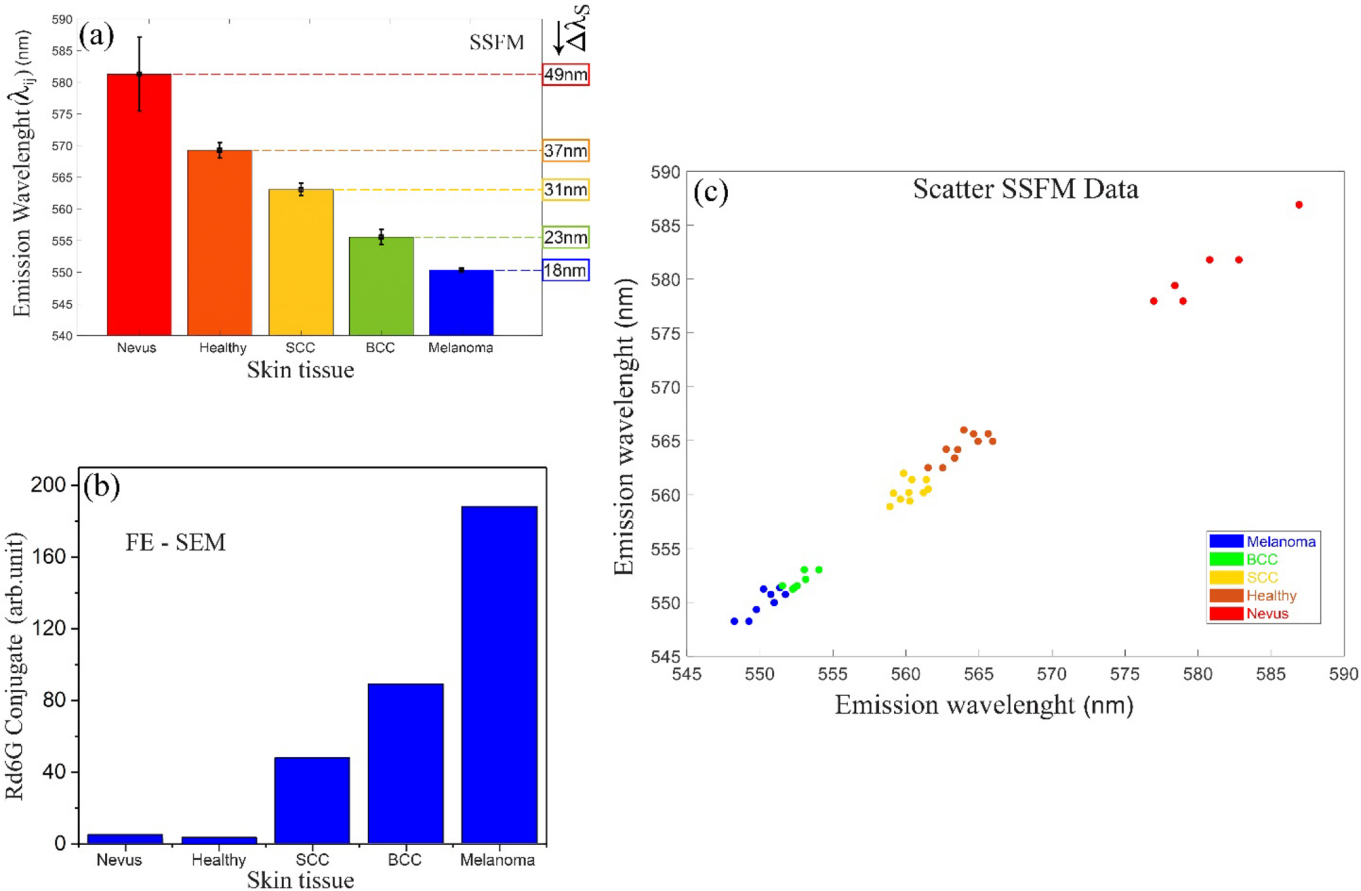
(**a**) Emission wavelength versus normal tissues/various malignant lesions based on SSFM micrographs. (**b**) Rd6G conjugates the attached fluorophores to the cells in stained melanoma, nonmelanoma, and normal/nevus tissues according to FE-SEM assessment. (**c**) Scatter plot of the specimens under the test indicating relative spectral shifts for each lesion type against normal tissues to determine the statistical significance of the diagnosis process, emphasizing a high confidence level due to lucid spectral segmentation and distinct spectral spacing among the tissue types.

Figure 5a depicts the relative population of active Rd6G stained in various samples from melanoma to healthy ones indicating the fluorophore congestion in descending order. Note that the pathologic discrepancies of nonmelanoma lesions arise from the local fluorophore concentrations. On the other hand, Fig. 5b illustrates a relative number of deactivated Rd6G fluorophores attached to the cells according to FE-SEM assessments. Those images indicate that the abundance of fluorophore conjugation/bondings favors each type of lesion against the normal tissues. Melanoma lesions contain heavy fluorophore conjugations to the cells highlighted in the blue color code for most pixels. Note that the emission wavelength peak ranges from 548 to 575 nm except for benign nevus (576–588 nm). Figure 5c shows the scatter data of all samples of interest, emphasizing a well-defined spectral segmentation. The spectral width corresponding to specimens’ data scattering is found to be 2 nm (melanoma), 4 nm (BCC), 3.3 nm (SCC), 4.1 nm (healthy), and 14.8 nm (nevus). Benign nevi undergo a larger spectral area characterized by the longest emission wavelength (largest redshift) over 576–588 nm ($\sim$ 12 nm width). The nevi spectral region is well-separated from normal/cancerous tissues because of the attendance of many melanocyte pigments within the nevi, which results in high amplitude accompanying larger spectral emission (width).

Pigmented nevi contain nevomelanocytic nevus cells derived from the neural crest that share with normal skin melanocytes to produce melanin^57^. The absorption spectra of melanin in skin vary wide from UV to visible spectral range^58^. Melanocytic nevi are benign neoplasms or hamartomas composed of melanocytes^59^. Nevus cells are a variant of melanocytes^60^, which include endogenic fluorophores to elevate the relative intensity values of fluorescent maxima^57^. The fluorescence spectra of skin with a high level of melanocytic nevi activated by green light excitation reveal the fluorescence emissions ranging from 550 to 700 nm with maxima $\sim$ 600 nm. Thus, melanin autofluorescence strongly affects the Rd6G fluorescence property leading to intense emission alongside a wide extreme redshift^61^.

According to RGB codes, the dominant blue, green, and yellow appear in neoplasia against red, featuring normal tissues. These lucidly attest to the SSFM competence to discriminate various cancerous lesions against healthy ones based on the solid Rd6G affinity to mitochondria. Eventually, in vivo SSFM is supposed to comfort the patients from the biopsy facilitating prompt diagnosis in the near future by choosing appropriate bio-compatible fluorophores with a high quantum efficiency. Furthermore, the endogenous fluorescence of the natural fluorophores of the skin could be helpful for in-vivo spectral imaging provoked by a suitable UV laser.

## Methods

### Apparatus

**Figure 6:**
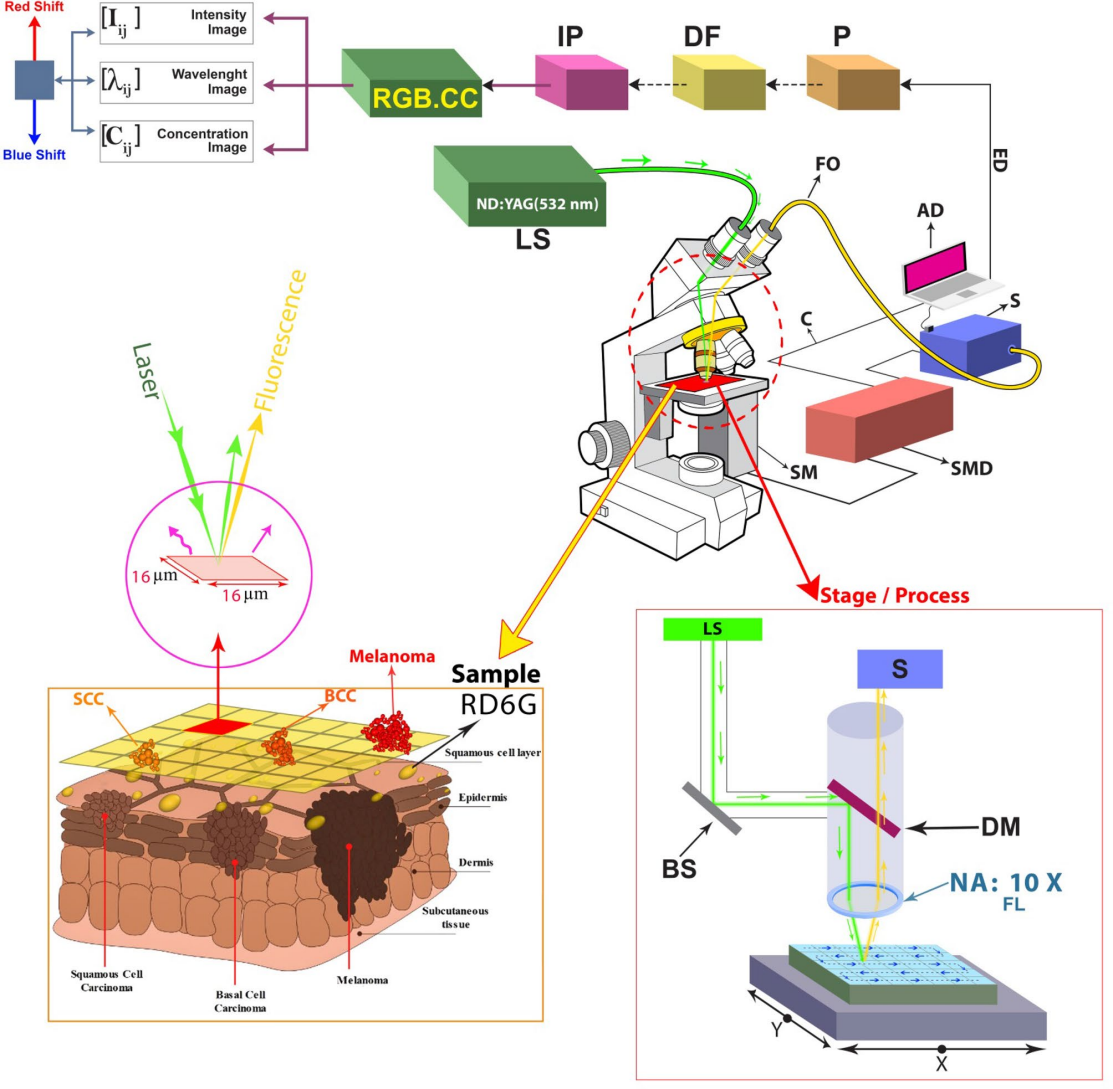
Schematic arrangement of SSFM and Rd6G concentration, including signal processing unit, filtering, and image processing unit, gives out three sets of fluorescence micrographs, i.e., signal intensity images, spectral micrographs, and Rd6G concentration mapping. (*IP* image processing, *DF* digital filter, *P* processing, *ED* exporting data, *AD* analysis data, *S* spectrometer, *SMD* step motor drive, *SM* step motor, *LS* laser source, *DM* dichroic mirror, *BS* beam splitter, *FL* focus lens, *FO* fiber optic, *C* cable, *RGB.CC* RGB color codes).

Figure 6 illustrates the experimental setup of the point-scanning spectral imaging system. The homemade instrument consists of the microscope, SHG-Nd:-YAG laser, Czerny turner spectrometer, several mechanical parts for coupling the spectrometer with the microscope, and optomechanical components to manipulate the light using an x–y scanning translator mount. A grating-based spectrometer, Avantes, Avaspec2048, 200–1100 nm spectral range, with 0.4 nm spectral resolution, is coupled to an upright (Labomed, USA) microscope. The microscope is equipped with a 2D motorized stage in x and y directions. The spatial resolution of the stages’ mechanical movement ranges ~ 3–16 μm even smaller resolution can be achieved by establishing a piezoelectric transducer (< 1 μm). However, the slit size affects the final resolution limited to ~ 10 μm.

A laser is employed at 532 nm and 50 mW to excite the samples through a 10× ocular lens. Note that other lasers at the selective wavelengths, such as light emitted diodes (LEDs), may be replaced for different purposes. Two objectives are also exploited to focus the laser beam on the target, collecting the backward fluorescence light. The field of view of 10× (and 40×) objectives correspond to spot sizes of 2400 μm (and 400 μm), respectively. Some optical components are pre-designed to illuminate a spot size as small as ~ 10 μm to select the emission of the middle part of the pixel, and avoid overlapping the other ones. The backward fluorescence is collected through the optical bundle fiber (400 μm in diameter SMA, ncore905-UV600/660) into the spectrometer via a collimating lens. A spatial filter discards the undesired peripheral emissions too. The signal is separated from the excitation light using a spectral filter (or dichroic mirror) to transmit only wavelength longer than 532 nm and block the laser beam. For instance, note that 581 nm (the characteristic fluorescence emission of nevus) is longer than 532 nm, so the laser beam at 532 nm is blocked and the wavelength longer than 532 nm, which arises from fluorescence emissions, are recorded by the spectrometer accordingly.

In practice, the homemade microscope objective is kept at a vertical distance of ~ 5 mm to launch the scanning mode. Two microscopic objectives are utilized to conduct the laser beam onto the target, and then a focusing lens is employed to collect the spectral fluorescence emission through the same objectives. Moreover, the Czerny–Turner spectrometer consists of an analog to digital (A/D) converter to record the spectral data digitally. The spectrometer and motorized stage are synchronized via a controller circuit. After each scanning step, the spectrometer receives a fluorescence spectrum via the fiber bundle of each pixel location. Subsequently, data is recorded using the link data port in the computer memory. The acquisition time for each scan depends on the dimension of the scanning area, and the spectrometer’s integration time is set to be ~ 1 ms. Many raw spectral data are processed offline utilizing a proper homemade algorithm to obtain the spectral image based on denoising, spatial, and spectral filtering to scale up the high signal-to-noise ratio.

Regarding the current prototype of SSFM, the image is divided into (m = 60 and n = 30), typically 1800 pixels covering a 1$$000\times$$ 500 μm area, such that each pixel size is set to be 16 µm. The pixel size can shrink down to 3 μm, improving the spatial resolution. The translation of the mount is carried out horizontally along x and vertically along y-axes in raster form to collect the fluorescence light pixel by pixel, retrieving the overall image at the end. The laser is kept stationary, and the mount, including the tissue specimens, is relatively moved across the objective lens as long as the scanning area is over. The pair ($${x}_{i},{y}_{j} )$$ indicates the position of each pixel as the building block of the micrographs, and the corresponding signal intensity and the emission wavelength are recorded for whole pixels. Eventually, three sets of matrices are obtained, i.e., the signal intensity $${[I}_{ij}]$$ and the emission wavelength [$${\lambda }_{ij}$$], as well as the concentration $$[$$c_*ij*_]. Moreover, denoising processing is also performed to mitigate unwanted signals. The Gaussian filter is applied to give out high-resolution images. Consequently, the matrices are processed according to the algorithms given in the supplementary data, and the corresponding $$[c$$_*ij*_] is retrieved in the virtue of $$\left[\overline{k }\right]$$ and $${[\lambda }_{ij}$$]. Unlike conventional fluorescence microscopy, which deals with emission signals based on camera capturing of the signal intensity, SSFM as a laser scanning spectral microscope utilizes an appropriate spectrometer to form the spectral image from fluorescence emissions. This facilitates the skin cancer diagnosis regarding the significant spectral data.

Furthermore, the alternative CLSM images are taken from Rd6G stained tissues using Eclipse Ti2-A, Nikon-Japan (0.1 μm resolution), and H&E images taken by Olympus CX31 microscope. Note that the SSFM unit can be coupled with CLSM to upgrade the system performance leading to the prompt diagnosis. FE-SEM (Sigma 300-HV-Germany-Zeiss) is exploited to record the corresponding micrographs delineating the effect of Rd6G local distribution over cancerous/normal tissues.

It is worth noting that the spectral absorbance of endogenous fluorophores in the organ, such as flavin, elastin, porphyrin, lipids, etc., are mostly in UV spectral range. Those are not excited with laser at 532 nm, which lies in the visible range. However, the endogenous fluorescence of the natural fluorophores of the skin could be helpful for in-vivo imaging in the future provoked by a UV laser.

### Sample preparation

This is to certify that all methods in this manuscript were carried out in accordance with relevant guidelines and regulations according to the declaration of Helsinki. Furthermore, it is worth noting that all the human materials are archived specimens taken from Razi Skin hospital, Tehran University of medical sciences. In addition, we have no access to confidential personal information of the patients except their gender, age, and types of infections.

Here, several skin cancerous tissues are prepared, such as nonmelanoma and melanoma against some healthy tissue and benign nevi. The tissues were received from the Razi Hospital, Tehran, Iran. The samples are excised from 30 male and 20 female Persian patients over 3 years of follow-up. The normal samples are selected around the malignant peripheral tissues according to the code of ethics for the surgeon. The thickness of the section ranges from ~ 5 to 10 μm. On the other hand, Hematoxylin and Eosin (H&E) are preferred for viewing cellular and tissue structure detail. This technique is traditionally examined in histology laboratories as a routine procedure, including paraffin blocks to illustrate nuclear detail in the cells. After microtome slicing, H&E is used to stain the tissues. Then typical micrographs of the tissues are then prepared by the histotechnique method and subsequently observed under an optical microscope. The Rd6G fluorophore is supplied from the Merck company. It is dissolved in deionized water to prepare a typical 16 μm concentration using the ultrasonic device, Bandlin model H-102-D. Once the solution is ready for testing, the tissues are immersed in the Rd6G solution to fulfill the staining process. Afterward, the stained specimens are examined using CLSM, SSFM, and FE-SEM to obtain various micrographs systematically.

### Imaging algorithm

Let’s assume a 2D array including a large number of $m\times n$ pixels where m and n denote the number of pixels in row and column of cartesian order, respectively. The size of each pixel is selected to be a few micrometers. It is essential to analyze thousands of pixels to create a micrograph at last. Let’s suppose two different episodes of local fluorophores, (i) bare fluorophore without tissue and (ii) fluorophore with tissue staining.

As a consequence, a couple of matrices attributing the signal intensity and emission wavelength of many pixels are obtained for the fluorescence mapping of specimens as below:

1 $$\left[{I}_{ij}\right]=\left(\begin{array}{ccc}{I}_{11}& {I}_{12}& \cdots \\ {I}_{21}& {I}_{ij}& \vdots \\ \vdots & \dots & {I}_{nm}\end{array}\right),$$

2 $${[\lambda }_{ij}]=\left(\begin{array}{ccc}{\lambda }_{11}^{p}& {\lambda }_{12}^{p}& \cdots \\ {\lambda }_{21}^{p}& {\lambda }_{ij}^{p}& \vdots \\ \vdots & \dots & {\lambda }_{nm}^{p}\end{array}\right).$$

Subsequently, the fluorophore concentration matrix is also derived from [${\lambda }_{ij}$] as follows:

3 $${[c}_{ij}]=\left(\begin{array}{ccc}{c}_{11}& {c}_{12}& \cdots \\ {c}_{21}& {c}_{ij}& \vdots \\ \vdots & \dots & {c}_{nm}\end{array}\right),$$

where correlation between ${[\lambda }_{ij}]\, and$ ${[c}_{ij}]$ is given by:

4 $${[c}_{ij}]=\left[\overline{k }\right]{[\lambda }_{ij}],$$

where

5 $$\left[\overline{k }\right]=\left(\begin{array}{ccc}{k}_{11}& {k}_{12}& \cdots \\ {k}_{21}& {k}_{ij}& \vdots \\ \vdots & \dots & {k}_{nm}\end{array}\right).$$

[$\overline{k }$] ascertains the $m\times m$ characteristic matrix. It is a unity matrix for bare fluorophore and a diagonal matrix in the case of stained tissue. Instead, the nonlinear correction matrix is obtained in favor of stained tissues. It is worth noting that $\left[\overline{k }\right]$ is a parametric measure of the medium of interest explaining the fluorophore characteristics based on tissue structure. However, Rd6G conjugates with cells alter the fluorescence distribution such that normal/cancerous take their characteristic matrices. According to mathematical formalism, [k] is a measure to correlate [λ] and [c], and it is an important matrix based on the tissue structure. As an example, a homogenous solution of Rd6G follows the simplified formalism as below:

$$\left[\overline{k }\right]=kI,$$

$$\left[c\right]=kI\cdot \left[\lambda \right],$$

where I and k are unit and diagonal vectors, respectively.

And [c] becomes simplified as a scalar, i.e., $c=k\lambda$. On the other hand, for non-homogenous staining of tissues, $\left[\overline{k }\right]$ is a non-diagonal matrix. So, $\left[\overline{c }\right]$ is quite different from $[\overline{\lambda }]$ for the healthy and malignant tissues regarding the corresponding red and blue shifts.

**Figure 7:**
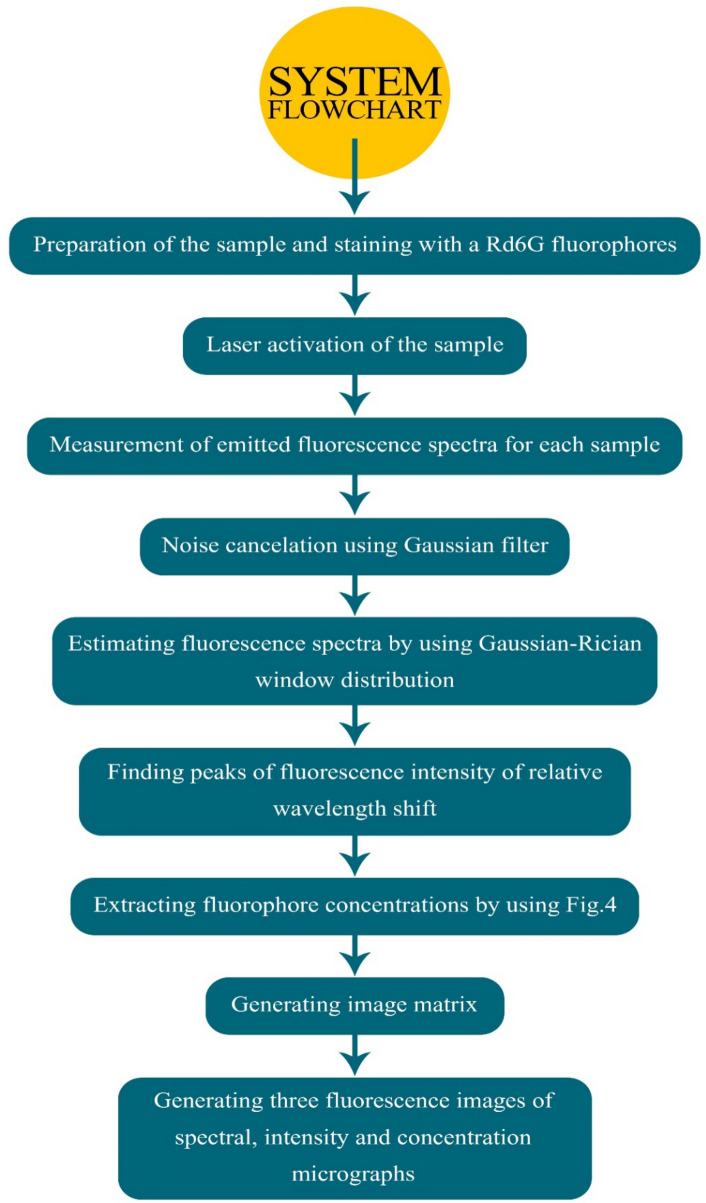
The stage-by-stage flowchart of the signal processing including tissue preparation, laser excitation, fluorescence spectra acquisition, denoising, data smoothing, and micrographs generation based on Figs. 1c and 4 data.

Figure 7 depicts the flowchart of the signal processing unit. At first, the preparation of the sample and staining with Rd6G fluorophores are carried out. Then, the specimen is stimulated by a proper laser. Afterward, the measurement of emitted fluorescence spectra is performed for each sample according to the imaging algorithm and based on m $\times$ n matrices. Then, a Gaussian filter is applied to the data to suppress the noise. Note that, to smooth the data, the Gaussian–Rician distribution is used. Meanwhile, the Gaussian–Rician window is employed in the fluorescence spectra to smooth the data. According to the measured data, the peaks of fluorescence intensity and the corresponding spectral shift are determined. The next stage is to generate the image matrices to retrieve the fluorescence micrographs.

After staining, the initial homogenous distribution of fluorophores drastically converts to an inhomogeneous distribution due to the morphology/pathology of the specimens. The spectra taken from normal and various cancerous tissues are examined by scanning the interest areas using the proposed SSFM. The discrepancies among normal and cancerous specimens carefully arise from the fluorophore conjugations with the cell mitochondria, whose affinity is drastically different from normal/cancerous tissues.

### Declarations

This manuscript is based on allowed patent with the application number of 16/706,748 and taken into account as one example application.

## Supplementary Information

Supplementary Information.

## Data Availability

All data are generated initially and analyzed during the prompt study and can be found in the article.
