# Supplementary material for: Discrimination of normal and cancerous human skin tissues based on laser-induced spectral shift fluorescence microscopy
Source: Sci Rep. 2022 Dec 3;12:20927. doi: 10.1038/s41598-022-25055-y (PMC9719548; doi:10.1038/s41598-022-25055-y)
Supplement: Supplementary file 1 — Supplementary Information. [file 41598_2022_25055_MOESM1_ESM.docx]

**Additional Information**

**Extended data**

Table.1 tabulates the three color codes for emission wavelength, signal intensity, and Rd6G concentration, respectively. The intensity color codes denote red R as strong emission, and blue B indicates faint signals. Typically, green G emphasizes median fluorescence intensity. For instance, R/B similarly demonstrates a high/low emission signals ratio ~6 such that R/G ≈ G/B ≅3. On the other hand, the spectral color codes categorize the normal/cancerous tissues based on the RGB order corresponding to the spectral shifts. A broad spectral range of 45 nm (540-585 nm) is under test regarding the peak fluorescence emission wavelengths in favor of various lesions and normal tissue except benign nevus, where spectral width extends up to 600nm. Similarly, R is the max spectral shift indicating the longest emission wavelength of $\sim$570nm for normal tissue, and B attributes the shortest emission wavelength at 548nm in melanoma. Consequently, G denotes the median color related to $\sim$555nm. Furthermore, R indicates the largest shift of 30nm, and B ascertains the minimum spectral shift with respect to the laser wavelength at 532nm. In addition, Rd6G concentration takes R as 16μM(0.77$\times{10}^{-2}$*g/lit)* and G$\sim$8μM (0.39$\times{10}^{-2}$*g/lit)* and B less than 1μM (0.096$\times{10}^{-2}$*g/lit*) in these series of experiments. Inevitably, the intensity, spectral, and concentration micrographs apply the same color codes but indicate different concepts.

**Extended data Table.1** Color codes addressing the emission wavelength, signal intensity, and Rd6G concentration distribution.


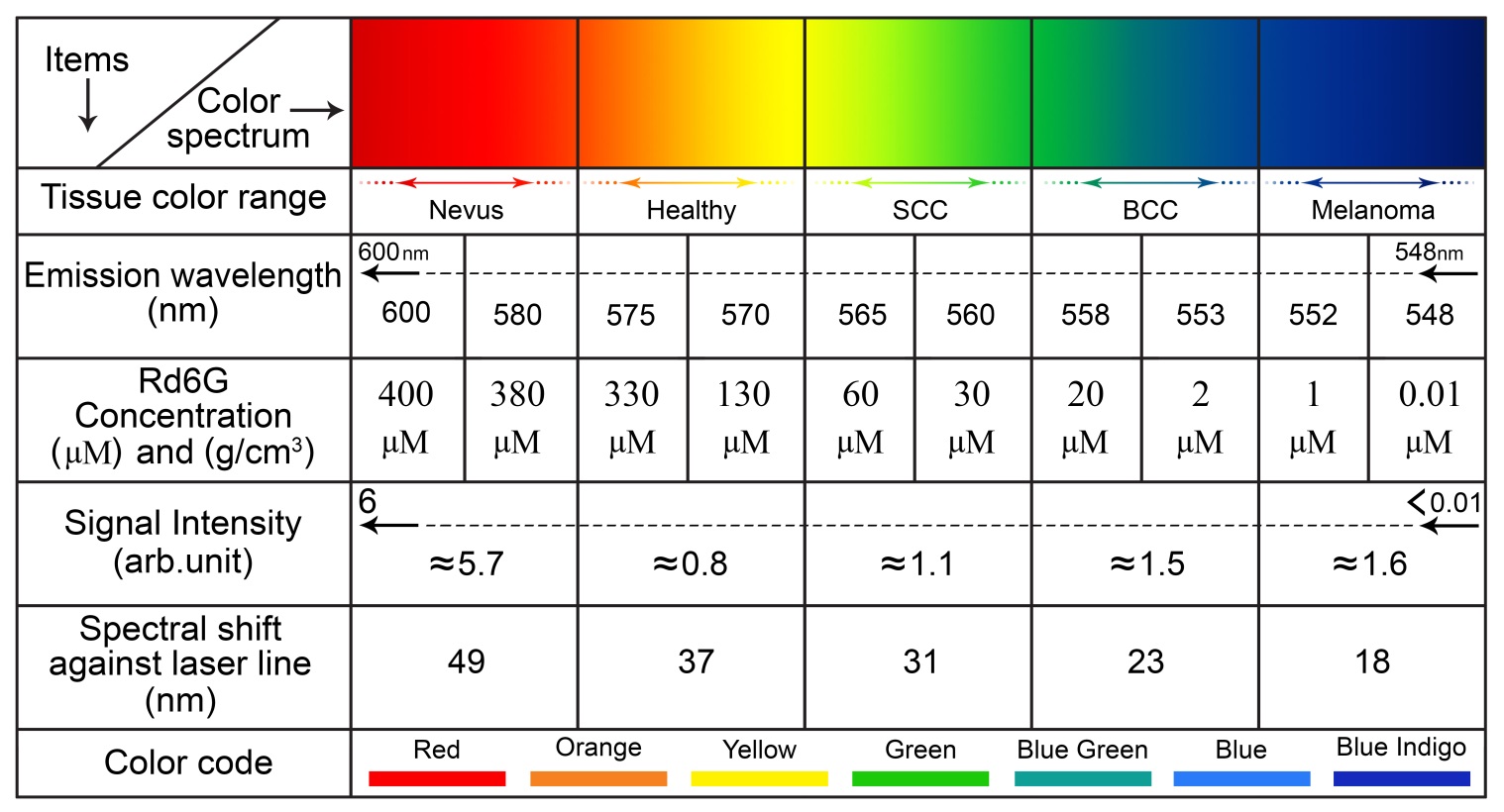


**
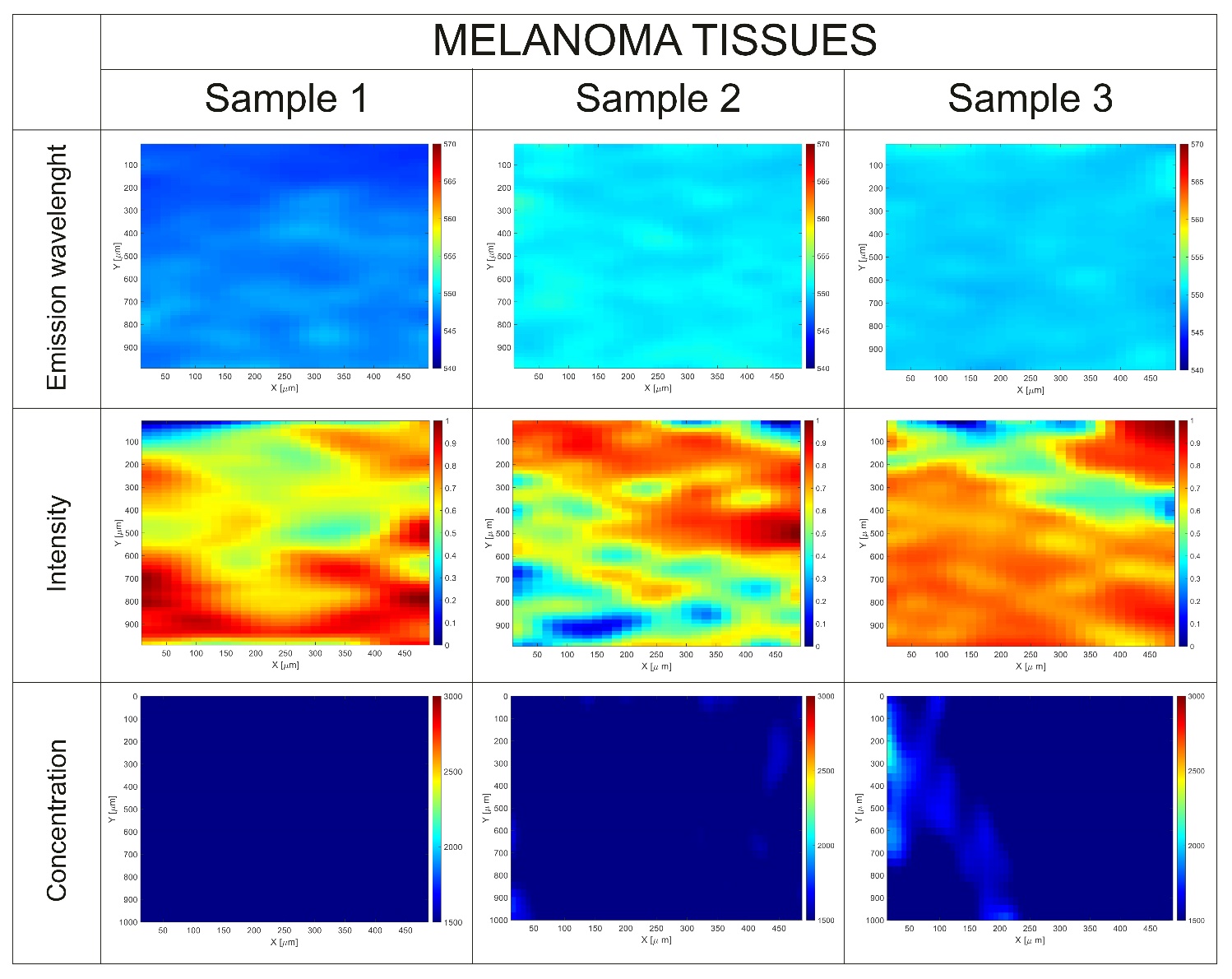
**

**Extended data Fig1.a** Typical SSFM micrographs. Top: emission wavelength, middle: signal intensity, down: Rd6G concentration for melanoma. The major feature of melanoma lesions is highlighted by a blue shift in spectral images and dominant intensity (red) alongside faint intensity signals.

Extended data Fig.1 illustrates three more micrographs of melanoma, BCC, SCC, healthy, and nevus, including top: emission wavelength, middle: intensity, and bottom: concentration micrographs.


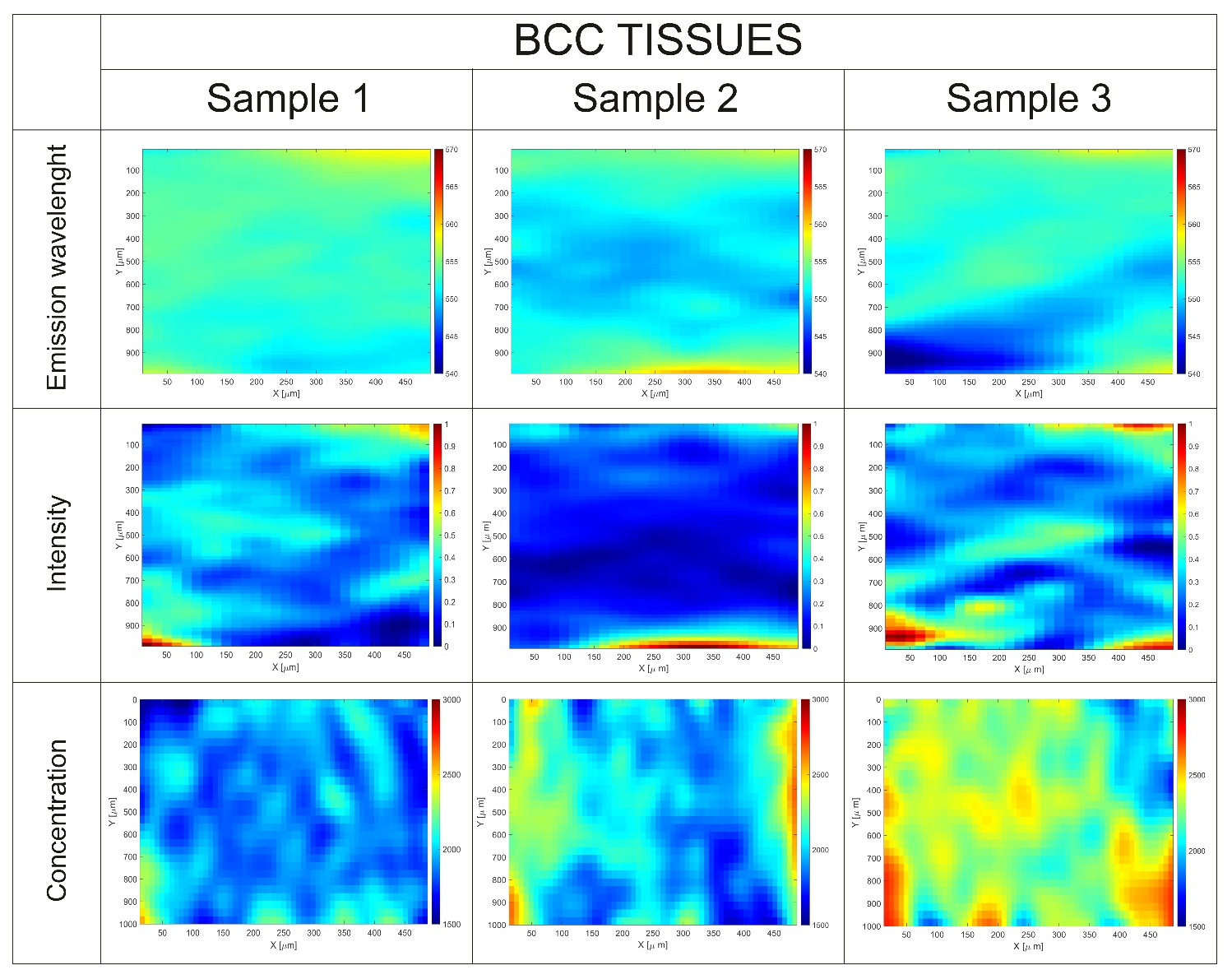


**Extended data Fig.1b** Typical SSFM micrographs. Top: emission wavelength, middle: signal intensity, down: Rd6G concentration for BCC tissues.

Furthermore, BCC tissues are characterized by blue-green color codes in favor of spectral micrographs and relatively faint intensity signals.


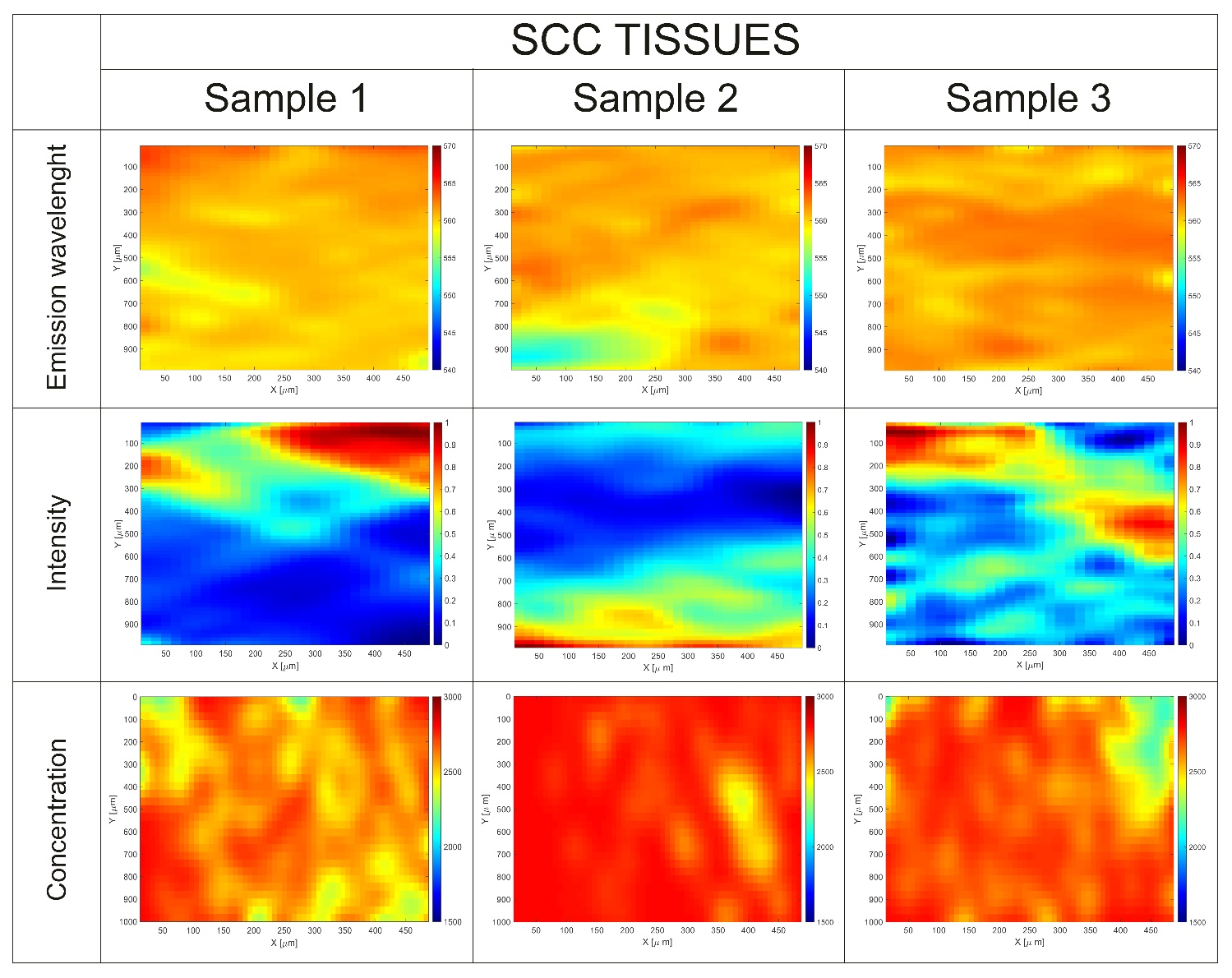


**Extended data Fig.1c** Typical SSFM micrographs. Top: emission wavelength, middle: signal intensity, down: Rd6G concentration for SCC tissues.

Similarly, SCC specimens take yellow-red color codes for emission wavelength images indicating a spectral shift to a larger wavelength and rather faint signals in the intensity micrographs. Similarly, concentration images more or less copy the emission wavelength micrographs.

**
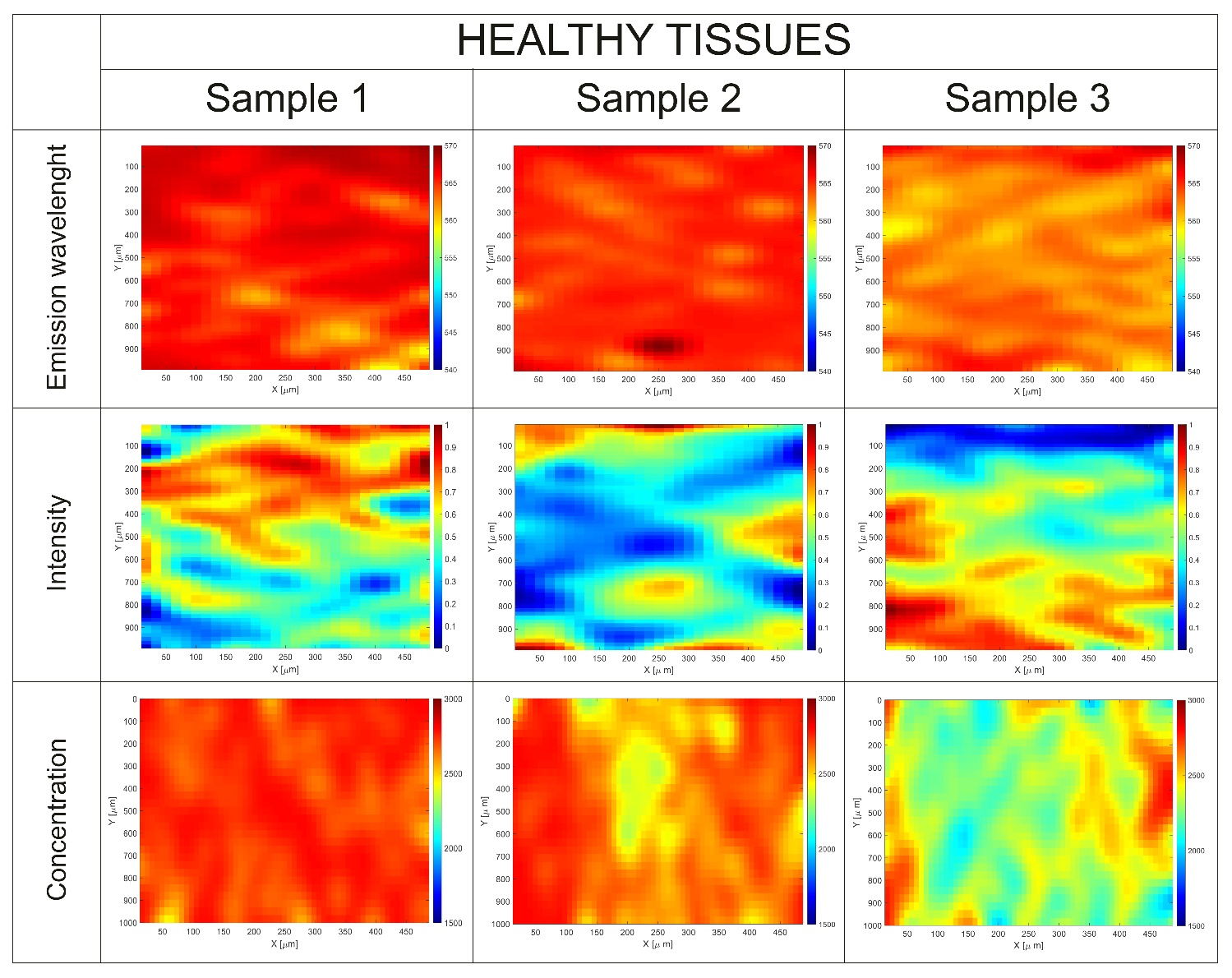
**

**Extended data Fig.1d** Typical SSFM micrographs. Top: emission wavelength, middle: signal intensity, down: Rd6G concentration for healthy tissues.

Healthy tissues approach red color codes regarding the spectral images alongside a mixture of faint/intense intensity signals. The concentration micrographs usually copy the spectral information as expected. However, those attest to the active fluorophore distribution over the tissues. A large number of active fluorophores are available throughout healthy and nevus samples, whereas those of melanoma and BCC lesions show excessive conjugation leading to a reduction of active fluorophores. SCC micrographs include a large abundance of fluorophores, featuring a red color code.

SSFM of nevus and healthy tissues are more or less similar. However, nevus spectral micrographs emphasize deep red color code indicating emission at a longer wavelength in comparison.

**
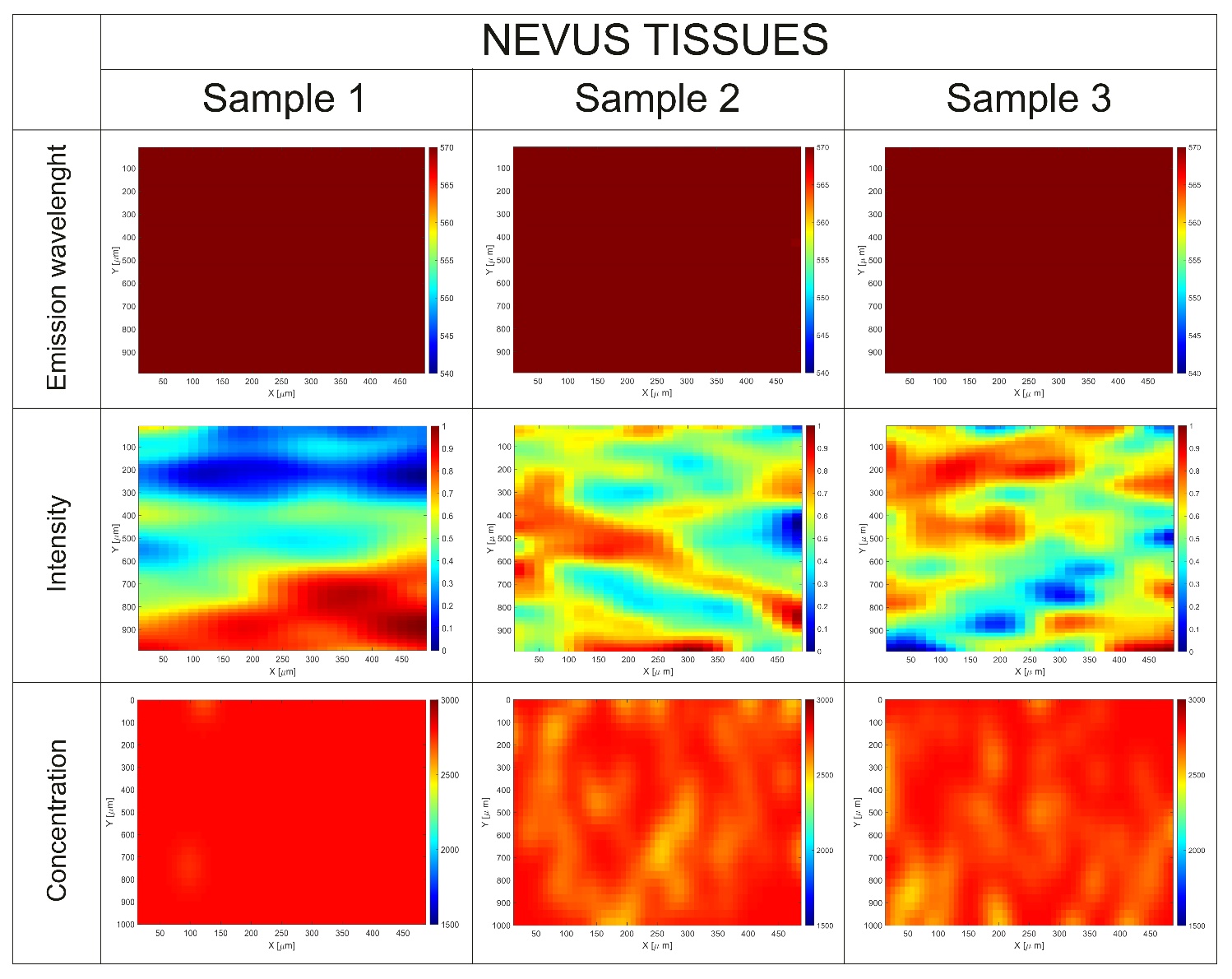
**

**Extended data Fig.1e** Typical SSFM micrographs. Top: emission wavelength, middle: signal intensity, down: Rd6G concentration for nevus tissues.

Extended data Fig.2a depicts the dominant fluorescence spectra of the measured data for each type of lesion. The peak amplitudes are compatible with the intensity images given in Fig1.b and Fig.4 (main text). The emission wavelength profile attests to a variation pixel by pixel along the length of all samples of interest. Spectral assessment includes mean value, standard deviation, and variation sequence to highlight the spectral profile. The statistical fluctuation along the pixels agrees with the intensity micrographs as shown in the extended data Fig.2.

Fig.2c shows the fluctuation around mean emission wavelength and the corresponding standard deviation pixel-by-pixel over typical tissue of all kinds. The standard deviation of melanoma data is relatively the smallest value. However, standard deviation increases for melanoma lesions and healthy sample take values, whereas it notably enlarges for nevus tissues. This arises from the attendance of many pigments as collective fluorescence emissions over a wide spectral range take place. Nevus has many pigments provoked by laser at 532nm, so the standard deviation is inherently much larger than healthy ones. In contrast, a single type of fluorophore (i.e., Rd6G) stains the malignant tissues. Note that the emission wavelength of the population of pigments is naturally much larger than a single type of fluorophore. According to Fig 2.a, Nevus, the device cannot detect emission signals longer than emission wavelength of 680nm. Thus, this is taken into account as the technical limitation of present SSFM.


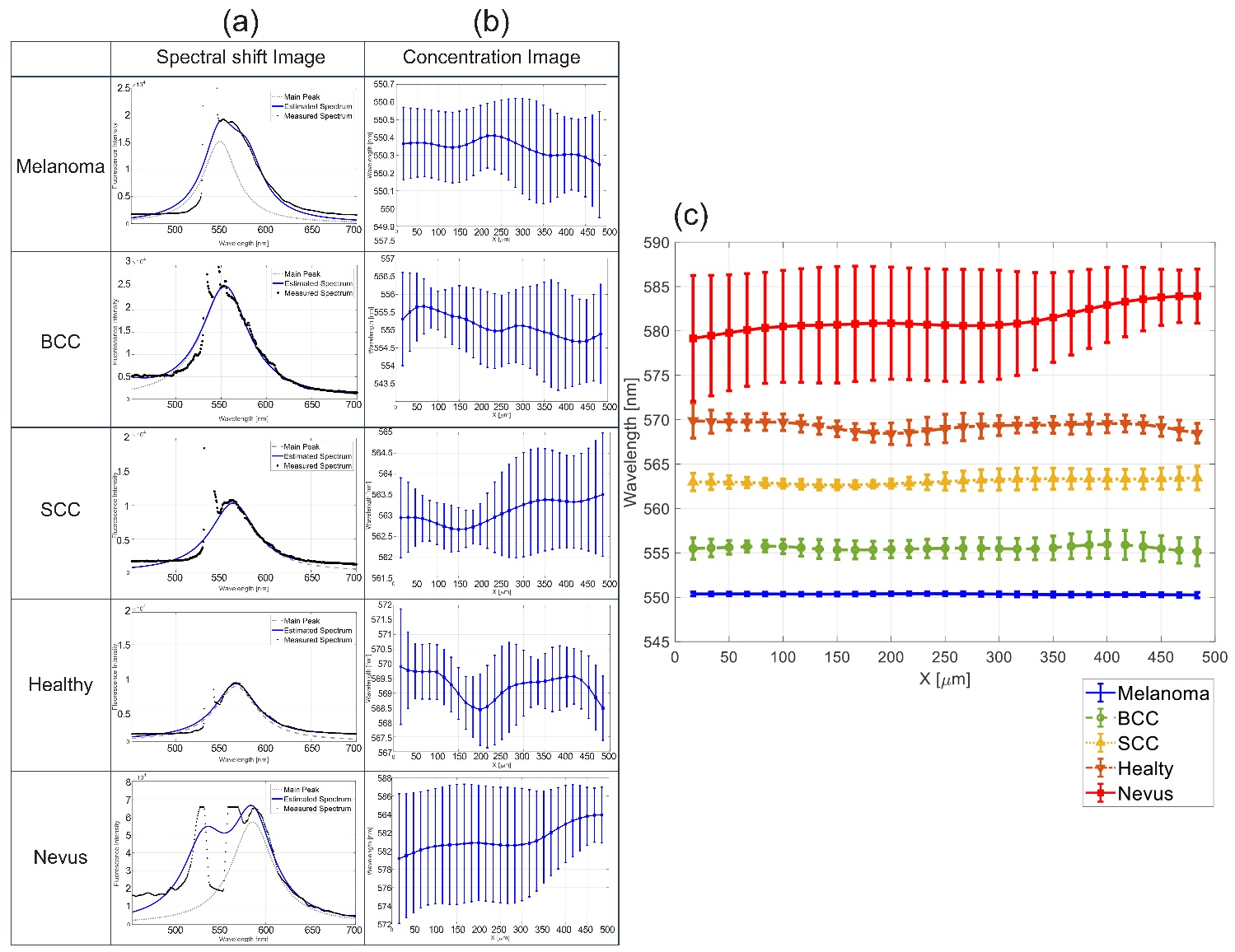


**Extended data Fig.2** a) Fluorescence spectra based on estimated spectra taking the average over all similar samples of interest to find the peak location the based on flowchart algorithm. Note that spectral shift images take color codes of blue, green/yellow, and yellow/orange corresponding to Melanoma, BCC, and SCC, respectively. The normal tissues take vivid red, and nevi are characterized by dark red. Nevus spectra are likely subject to some errors. Due to the technical limitations, SSFM can not detect emission signals longer than 680nm. b) Emission wavelength variation over pixels along the X-direction. c) The emission wavelength statistics over all pixels in the cancerous and healthy micrographs.

Eventually, extended data Fig.3a plots the scatter data over emission wavelength, including spectral shift with respect to laser line ($\Delta\lambda_{s})$and spectral scattering ($\Delta)$ for each type of lesion. Extended data Fig.3b also demonstrates the spectral width in favor of emission wavelength for the skin tissues of interest. The spectral discrepancy between melanoma/BCC and SCC/healthy and nevi reveal the statistical significance and confidence level of SSFM performance according to the sample population of any kind under test.


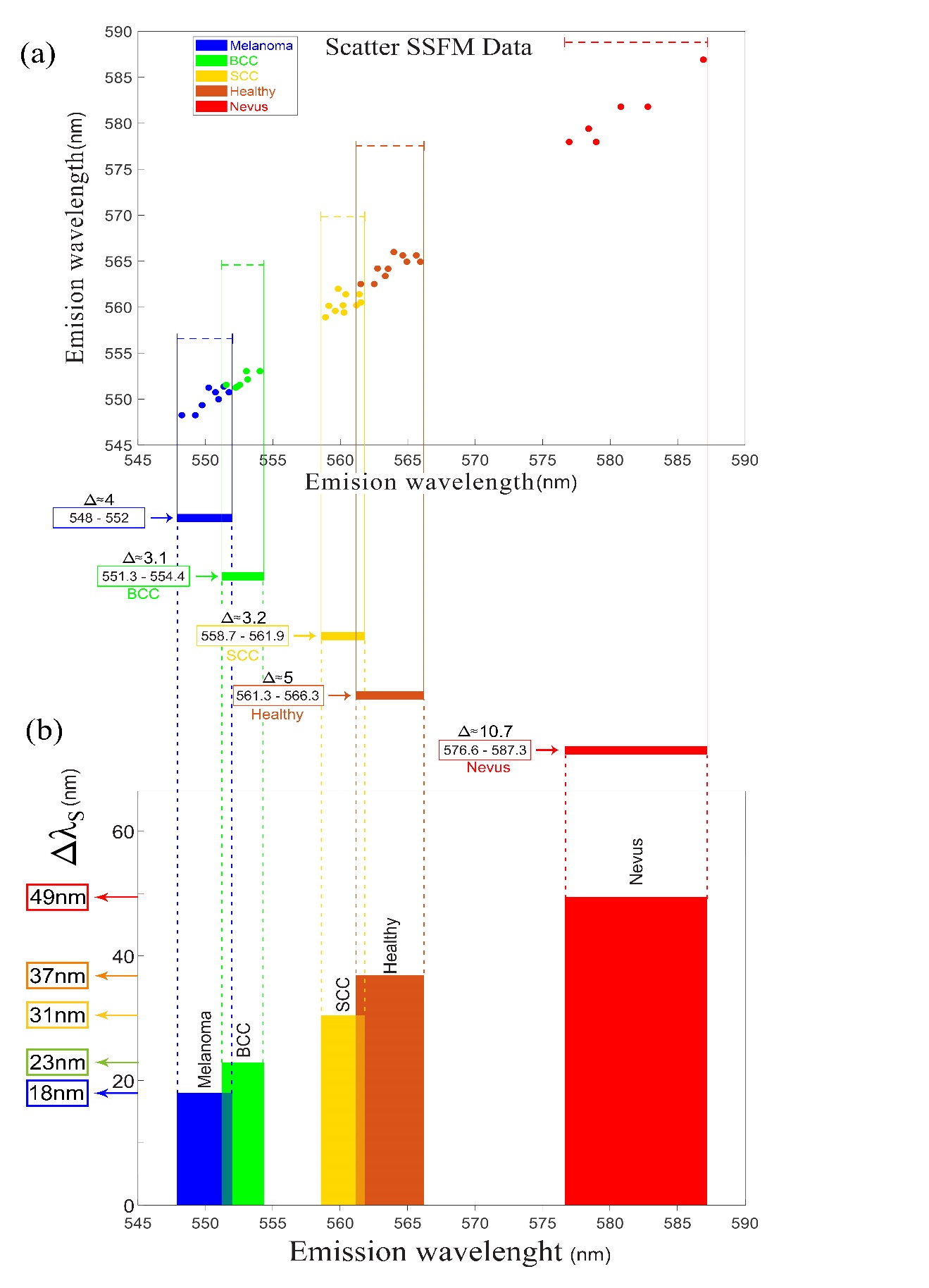


**Extended data Fig.3** a) Scatter data b) Spectral shift and spectral width over different malignant lesions and healthy tissues. Note that ($\Delta\lambda_{s})$ ascertains spectral shift with respect to the laser line. Furthermore, $\Delta$ denoted to be spectral scattering accordingly.

**Statistical analysis**

| **Paired Samples T-Test (P-value)** | **Paired Samples T-Test (P-value)** | **SD** | **Mean** | **Sample** |  |
| --- | --- | --- | --- | --- | --- |
| **Melanoma/Healthy** | **Melanoma/BCC** | 1.78308 | 549.845 | **Melanoma** | 1 |
| **0.001** | **0.049** |  |  |  |  |
| **BCC/Healthy** | **BCC/SCC** | 1.07146 | 554.9975 | **BCC** | 2 |
| **0.0001** | **0.001** |  |  |  |  |
| **SCC/Healthy** | **SCC/Healthy** | 0.5408 | 560.74 | **SCC** | 3 |
| **0.003** | **0.003** |  |  |  |  |
| **Nevus/Healthy** | **Healthy/Nevus** | 0.86079 | 564.915 | **Healthy** | 4 |
| **0.006** | **0.007** |  |  |  |  |
|  |  | 3.92823 | 581.515 | **Nevus** | 5 |

**Extended data Table.2** Scatter data of all samples of interest alongside the statistical assessment via SPSS software to determine the mean, SD, and P-value and the confidence level regarding the lesions population of different kinds and those of infiltrated (healthy) and benign nevi ones. Note that the spectral scattering for emission wavelengths in each category of tissues are given to be melanoma (4nm), BCC (3.1nm), SCC (3.2nm), healthy (5nm), and nevus (10.7nm). Table.2 tabulates the P-value of the pair of tissue lesions according to the SPSS analysis, which attests to the high accuracy in the SSFM performance. However, some errors may come from the peripheral adjacent malignant infiltrated tissue. Moreover, the time-consuming biopsy and preparation method may induce extra errors.

A probability value (p-value) or statistical significance is a statistical measurement used to validate a hypothesis against observed data which measures the probability of obtaining the observed results, assuming that the null hypothesis is true. Here, the Statistical Package for the Social Sciences (SPSS) software is used to assess the p-values of the different groups of tissues using paired samples T-test analyzing tool. Table.2 tabulates the P-value of a pair of different sets of tissues, i.e., melanoma-BCC and BCC-SCC, and SCC-healthy and healthy-nevus, indicating a high confidence level of 95$\%$, corresponding to a P-value of 0.049, 0.001, 0.003 and 0.007, respectively. All results are statistically significant P-value $<$0.05. Furthermore, the P-values of all the lesions against healthy specimens are tested to assume the SSFM performance. In addition, we have calculated the p-values for all the lesions and healthy samples using SPSS software. This attests that those pairs are statistically significant.
